# State-of-the-Art Macromolecules in Portugal

**DOI:** 10.3390/ijms25010620

**Published:** 2024-01-03

**Authors:** Artur J. M. Valente, Nuno C. Santos, Maria João Moreno

**Affiliations:** 1 University of Coimbra, Department of Chemistry, 3004-535 Coimbra, Portugal; avalente@ci.uc.pt; 2 Instituto de Medicina Molecular, Faculdade de Medicina, Universidade de Lisboa, 1649-028 Lisbon, Portugal

This Special Issue presents five contributions covering various topics, as it would be expected for an area as comprehensive and multidisciplinary as Macromolecules. The broad diversity of the research work developed at this level in Portugal on the intersection between biological sciences, chemistry, and physics includes studies of proteins and polysaccharides, their interaction with lipid membranes and nanoparticles, and their impact on cell features relevant to health and disease.

Khalil et al. presented a review on the inter-relation between cell shape, cell function, and the occurrence of diseases, with a particular emphasis on cancer. The review is based on the processes in which normal and pro-tumoral cells can break the constraints imposed by cell shape and progress to a transformed phenotype. The article is organized into three complementary sections, namely: how knowledge on the organization and the dynamics of cytoskeletal macromolecules allows for the control of cell shape; representative cases of changes in cell shape and how they can alter the transcriptional program; and, finally, how cell shape could be utilized to prevent tumorigenesis.

Another review article included in this Special Issue, by Moreno et al., provides a comprehensive discussion of the methods used to assess the equilibrium distribution of ligands (small molecules) in the presence of complex systems, including proteins and/or lipid membranes. It is far from easy to obtain accurate information regarding ligand binding sites, affinity, and stoichiometry. Thus, the elucidation of the mechanisms involved in such complex heterogeneous systems is of notable importance, as they are closely related to biological activity. However, the authors’ discussion goes beyond providing a mere description of the methodologies, pointing out the limitations of some approaches and misanalyses. The authors present a new model for the quantitative evaluation of the binding affinity of ligands, based on partition coefficients, which is of particular relevance in the case of membrane proteins.

The paper written by Serrasqueiro et al. reports on the functionalization of chitosan–fucoidan nanoparticles with mannose and mannan, followed by biochemical studies of these conjugates. In this study, nanoparticles were prepared by coacervation, and functionalization was achieved by coating the nanoparticles with carbohydrates. The nanoparticles obtained showed a monodispersed size distribution, with diameters ranging from 200 to 500 nm. The effect of these nanoparticles on macrophage activation was also evaluated. This activation is, as an example, a crucial step in the induction of adipose tissue inflammation, being influenced by adaptive immune cells [1]. The assessment of macrophage activation commonly relies on the measurement of the production of cytokines and other secreted products [2]. The testing of toxicity and the internalization of nanoparticles was performed through the use of THP-1 monocytes and THP-1-differentiated macrophages. The authors demonstrated that the use of carbohydrate-containing chitosan-based nanoparticles led to macrophage activation, as determined by the production of the pro-inflammatory cytokines interleukins 1β and 6 and tumor necrosis factor-α, proving their potential for therapeutic applications.

Two other contributions were based on the measurement of the mutual interdiffusion coefficients for the assessment of interactions in systems of biological and biomedical relevance.

The first of these papers, by Fangaia et al., reports the effect of chlorhexidine digluconate on the elution of cobalt and chromium ions in artificial saliva media. These metal ions are present in dental alloys and, over time, tend to diffuse into the mouth. It was found that there was a strong interaction between digluconate and Co^2+^ and Cr^3+^ ions (as also observed through the use of UV-Vis spectroscopy). These results suggest that digluconate-containing mouthwashes may contribute to reducing toxicity levels in the oral cavity.

The second of the aforementioned contributions focuses on the interactions between β-cyclodextrin and sodium hyaluronate (243 kDa). The latter has an important role in lubricating and transporting nutrients to, for example, mobile joint structures of the human body. The study assessed how the cyclodextrin interacts with sodium hyaluronate and thus contributes to the improvement of mechanical and chemical stability properties. It was found that in the presence of cyclodextrin, a significant decrease in the viscosity of hyaluronate salt was observed, suggesting that this cyclodextrin may induce the aggregation of the hyaluronate by acting as a crosslinker. This is also supported by analysis of the ternary intermolecular diffusion coefficients. The interplay between diffusion and viscosity prevailed, and hyaluronate–hyaluronate interactions could be controlled through the addition of β-cyclodextrin.

The publication process of the manuscripts included in this Special Issue followed all best practice procedures. Independent editors were always selected whenever a guest editor was among the co-authors or had any present or past relation with the authors of the manuscripts submitted. Manuscript revision was always performed by independent reviewers, with the revision reports and the authors’ answers being available on the respective publication webpage. The manuscripts took between 22 to 35 days from submission to be accepted, with an average publication timeline of 27 days. The number of self-citations was 17% on average, with all publications being within the limit of 15%, except for one of the review articles, due to the inclusion of many authors with expertise in the different aspects considered. The works cited by the publications included in this Special Issue cover all relevant publishers in the field, with only 6% of the cited articles originating from journals published by MDPI. For the remaining cited articles, 31% originated from publications from Elsevier, 8% from ACS, 8% from Springer, 5% from Cell Press, 5% from Springer Nature, and 37% from 62 additional publishers, with each one of them corresponding to less than 5%.

It is our hope that you will enjoy reading some of these examples of state-of-the-art research on Macromolecules conducted in Portugal.
